# Identifying changes in dynamic plantar pressure associated with radiological knee osteoarthritis based on machine learning and wearable devices

**DOI:** 10.1186/s12984-024-01337-6

**Published:** 2024-04-03

**Authors:** Gege Li, Shilin Li, Junan Xie, Zhuodong Zhang, Jihua Zou, Chengduan Yang, Longlong He, Qing Zeng, Lin Shu, Guozhi Huang

**Affiliations:** 1 Department of Rehabilitation Medicine, Zhujiang Hospital, Southern Medical University, Guangzhou, China; 2 School of Rehabilitation Medicine, Southern Medical University, Guangzhou, China; 3 Department of Rehabilitation Medicine, the First Affiliated Hospital of Nanchang University, Nanchang, China; 4 School of Microelectronics, South China University of Technology, Guangzhou, China; 5 Department of Clinical Medicine, Xiamen Medical College, Xiamen, China; 6 School of Future Technology, South China University of Technology, Guangzhou, China; 7 Pazhou Lab, Guangzhou, China

**Keywords:** Knee osteoarthritis, Machine learning, Wearable device, Gait analysis, Plantar pressure

## Abstract

**Background:**

Knee osteoarthritis (KOA) is an irreversible degenerative disease that characterized by pain and abnormal gait. Radiography is typically used to detect KOA but has limitations. This study aimed to identify changes in plantar pressure that are associated with radiological knee osteoarthritis (ROA) and to validate them using machine learning algorithms.

**Methods:**

This study included 92 participants with variable degrees of KOA. A modified Kellgren–Lawrence scale was used to classify participants into non-ROA and ROA groups. The total feature set included 210 dynamic plantar pressure features captured by a wearable in-shoe system as well as age, gender, height, weight, and body mass index. Filter and wrapper methods identified the optimal features, which were used to train five types of machine learning classification models for further validation: k-nearest neighbors (KNN), support vector machine (SVM), random forest (RF), AdaBoost, and eXtreme gradient boosting (XGBoost).

**Results:**

Age, the standard deviation (SD) of the peak plantar pressure under the left lateral heel (f_L8PPP_std), the SD of the right second peak pressure (f_Rpeak2_std), and the SD of the variation in the anteroposterior displacement of center of pressure (COP) in the right foot (f_RYcopstd_std) were most associated with ROA. The RF model with an accuracy of 82.61% and F1 score of 0.8000 had the best generalization ability.

**Conclusion:**

Changes in dynamic plantar pressure are promising mechanical biomarkers that distinguish between non-ROA and ROA. Combining a wearable in-shoe system with machine learning enables dynamic monitoring of KOA, which could help guide treatment plans.

**Supplementary Information:**

The online version contains supplementary material available at 10.1186/s12984-024-01337-6.

## Background

Osteoarthritis (OA) is a chronic, irreversible disease characterized by degeneration of the hyaline articular cartilage, subchondral bone, ligaments, capsule, synovium, and periarticular muscles [1, 2]. Over 300 million people worldwide suffer from OA [3], and knee osteoarthritis (KOA) accounts for approximately 85% of the burden [4]. However, most cases of OA are not appropriately managed [1]. KOA can result in substantial economic losses [5]. In addition, individuals with KOA may be debilitated by pain, stiffness, decreased range of motion, and gait dysfunction [6, 7]. As an age-related disease, KOA is a leading cause of pain and disability among the elderly [6]. Dynamic function tests such as the 40-m (4 × 10 m) fast-paced walking test and timed up and go test are recommended by the Osteoarthritis Research Society International [8]. The incidence of KOA cases is on the rise due to the global population aging and the increasing prevalence of obesity [6], thereby exacerbating the socioeconomic burden associated with this disease. Thus, examinations in different dimensions are essential for effective treatment of KOA.

Imaging examinations like radiography can be used to diagnose OA, grade OA severity, assess OA progression, and monitor treatment responses [9]. Radiography is the first choice in the clinical setting [10]. Radiological OA (ROA) is defined as a case whose Kellgren–Lawrence (K–L) grade is ≥ 2 and indicates that there are definite changes in joint structure. Although the semi-quantitative K–L grading system is widely used in clinical settings [11], it only reflects the static joint structure and requires large equipment and specialized professionals. Exploring additional dynamic features related to radiodiagnosis, such as plantar pressure during walking can enhance assessment tools and offer a new perspective for treatment management.

Gait function, an important indicator of rehabilitation, declines with the progression of KOA. Compared with the Western Ontario and McMaster University Osteoarthritis Index (WOMAC) and the Knee Injury and Osteoarthritis Outcome Score, quantified gait parameters are more objective for functional assessment of KOA patients [12]. Normal individuals and KOA patients can be distinguished by assessing gait parameters such as center of pressure (COP) [13–15], regional pressures [13, 16], contact area [16], and peak pressure [15, 16]. Plantar pressure can be measured using a force platform or an in-shoe system (e.g. pedar-X system). Force platforms are typically limited to controlled settings like hospitals and laboratories, whereas in-shoe systems, which comprise pressure sensors and Bluetooth transmission devices, provide a clear advantage in terms of flexibility, efficiency, and portability [17, 18]. Moreover, optimal shoes for patients with KOA remain unknown [19–21]. Changes in plantar pressure for patients with KOA are one of the important factors to consider when designing modified footwear. Thus, how dynamic plantar pressure changes in patients with KOA should be further investigated, which may provide guidance for the optimal modified footwear.

Machine learning uses algorithms that learn from data and make decisions with minimal human intervention [22]. Implementing machine learning in wearable devices will allow long-term and dynamic monitoring, which has already been used to detect plantar fasciitis [23], assess fall risk [24, 25] and stroke rehabilitation [26], and monitor freezing of gait in patients with Parkinson’s disease [27]. Although Wang et al. [28] used wearable insoles and machine learning to detect KOA, the progression of KOA could not be characterized in the study population, which included patients scheduled for surgery due to ineffectively conservative treatment as well as healthy subjects without symptoms of KOA. Other studies developed machine learning models to classify patients based on K-L grades [29] and WOMAC scores [30] using other gait parameters (e.g. spatial–temporal parameters) captured by force plates and three-dimensional optical motion capture systems. However, wearable devices and machine learning are seldom used to classify the severity of KOA based on plantar pressure distributions.

This study aimed to identify changes in dynamic plantar pressure associated with non-ROA and ROA using wearable devices and machine learning models. We hypothesized that dynamic plantar pressure measurements would significantly change following structural alterations of knee (i.e. ROA). This study offers insights into the dynamic monitoring of KOA and also guides the rational design of modified footwear to improve treatment outcomes for patients with KOA.

## Methods

### Participants

All participants were enrolled between January 2022 and February 2023 from the Pearl River Osteoarthritis Cohort (PROC) study at the Zhujiang Hospital of Southern Medical University, Guangzhou. This study was approved by an Institutional Review Board (IRB No. 2019-KY-016-02), and informed consent was obtained from all participants. The PROC includes people aged 45–79 years old from OA outpatient clinics and the communities nearby, who have never had knee surgery but reported having experienced knee pain most of the time in the past month. Exclusion criteria in this study were as follows: (1) with a history of spine surgery and severe spine disease (e.g. radicular pain to the lower extremity); (2) hip, ankle, or foot arthritis; (3) foot deformity (foot varus/valgus, flatfoot, etc.); (4) inability to walk at least 100 m without assistance; (5) acute knee injury in the past year. The calculated methods of the total sample size were as follows: (1) the sample size of the training set should be at least ten times of the included features; (2) the sample size ratio of training set to testing set was 3:1.

### Data collection

**Fig. 1:**
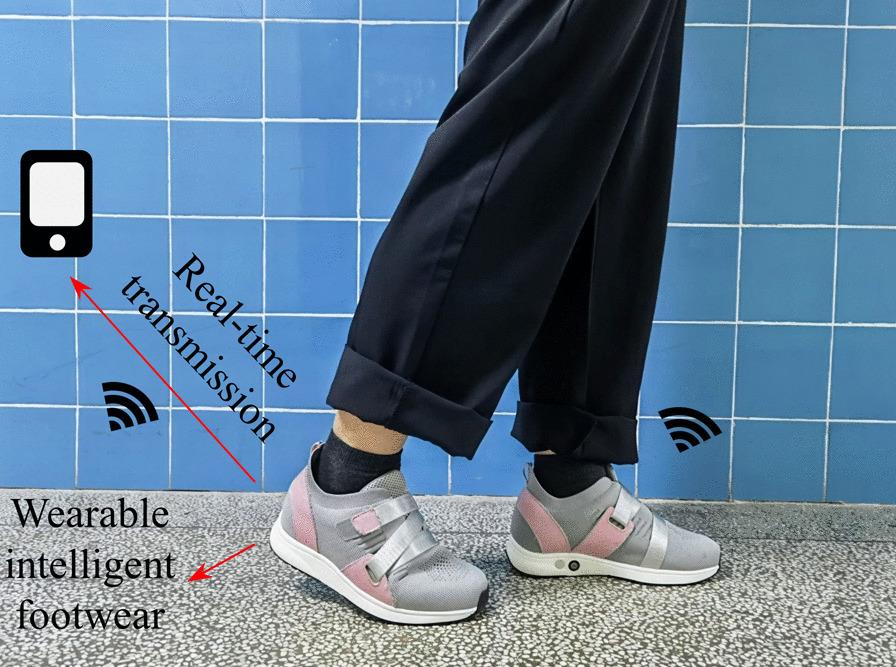
Wearable in-shoe system

A wireless footwear system (Fig. 1) with a sampling frequency of 20 Hz was provided by the South China University of Technology and used to acquire plantar pressure signals. An insole with a pressure sensor array and data acquisition unit was embedded in the footwear. Sensors were distributed across eight regions: the great toe (sensor 1), the first metatarsal head (sensor 2), the second and third metatarsal head (sensor 3), the fourth and fifth metatarsal head (sensor 4), the medial midfoot (sensor 5), the lateral midfoot (sensor 6), the medial heel (sensor 7), and the lateral heel (sensor 8). The sensor positions mentioned above were determined based on previous research [15]. The stability and effectiveness of the footwear system have been validated by previous studies [24, 31, 32]. The participants were asked to wear socks and appropriate shoes. To reduce deviations from their natural gait, participants walked freely for three minutes before the measurements began. In the formal walking stage, the participants were asked to walk 60 m (three times back and forth on a 20-m straight level walkway). Real-time data were transmitted wirelessly via Bluetooth and displayed on a smartphone.

Bilateral anteroposterior radiographs were acquired while the participants were weight-bearing in a double-leg stance (DLS) at a knee flexion angle of 20°–30°. ROA was diagnosed by an experienced radiologist according to the modified K–L (mK–L) grading system [33, 34]. ROA was defined as mK–L grade ≥ 2; all other cases were defined as non-radiological knee OA (non-ROA). Additional file 1: Table S1 describes the mK–L grading system. Radiographs of 20 randomly selected cases were assessed by another experienced radiologist; the intra-observer (κ = 0.814) and inter-observer (κ = 0.827) reliabilities were good.

### Data preprocessing

Abnormal and unstable data were discarded according to the midgait method [35] and the bimodal characteristics of plantar pressure. Sixty valid gait cycles were obtained from each participant after segmentation and splicing.

### Feature extraction

The extracted multidimensional features of dynamic plantar pressure were as follows: the peak plantar pressure (PPP), the pressure gradient (PG), time-domain features, impulse, the medial–lateral (M/L) plantar pressure ratio, the COP, the symmetry index, and the mean and standard deviation (SD) of the above parameters in 60 valid gait cycles. Methods used to calculate dynamic plantar pressure are described in Additional file 1: Method S1. The total plantar pressure feature set included 210 features (Additional file 1: Table S2). Physiological features related to KOA: age, body mass index (BMI), gender, height, and weight were also included in the feature selection.

### Feature selection

Selecting effective feature subsets from the total feature set can reduce overfitting and increase generalizability of the model. The Shapiro–Wilk test was used to assess the normality of the data. The Mann–Whitney U test and independent t-test were used to classify features that did not significantly differ between groups. Statistical significance was defined as a *p-*value < 0.05. A wrapper method was used to further optimize the selection of features among different groups.

### Machine learning model training and evaluation

**Fig. 2:**
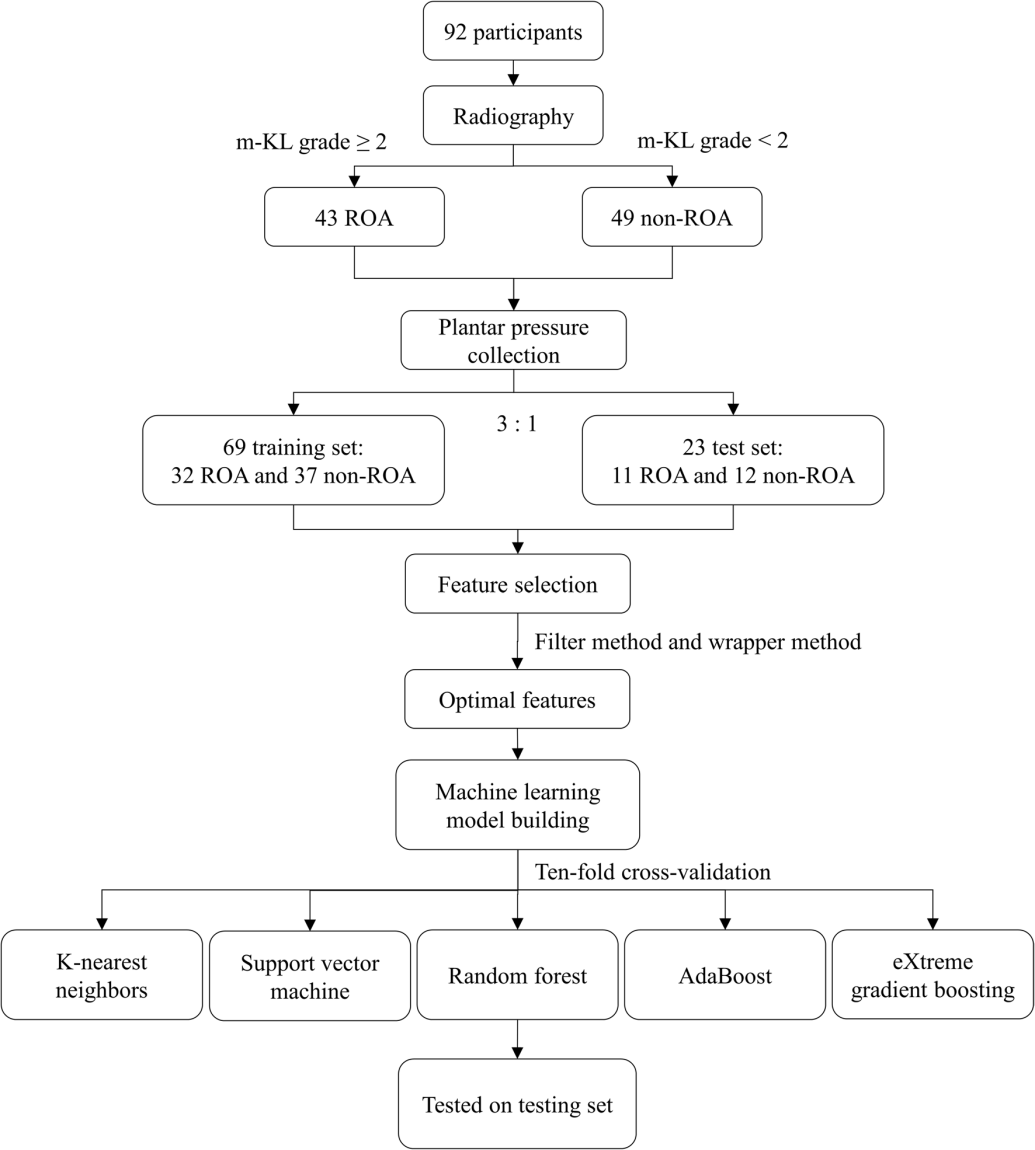
Flowchart of model building

The optimal features were used to train five types of machine learning algorithms including k-nearest neighbors (KNN), support vector machine (SVM), random forest (RF), AdaBoost, and eXtreme Gradient Boosting (XGBoost). Ten-fold cross-validation was implemented to train and evaluate the models, whose performance was measured based on accuracy and F1 scores obtained from the confusion matrix. Figure 2 outlines the methodology used to build the model. This study used Python 3.10.8 for model training and testing. The Optuna framework was used for hyperparameter tuning, which is an automated hyperparameter tuning software framework specifically designed for machine learning algorithms using Python programming.

## Results

### Participant characteristics

A total of 92 participants were recruited, of whom 43 were classified as ROA and 49 as non-ROA. Their demographic characteristics are shown in Table 1. The ROA group was older (*p* = 0.018) and had a larger BMI (*p* < 0.001) on average.

**Table 1:** Comparisons of participants' characteristics

Characteristics	Total (n = 92)	Non-ROA (n = 49)	ROA (n = 43)	*p*
Gender (n, %)				0.977
Male	17 18.5%)	9 (9.8%)	8 (8.7%)	–
Female	75 (81.5%)	40 (43.5%)	35 (38.0%)	–
Side of involvement (n, %)				0.067
Left	50 (54.3%)	31 (33.7%)	19 (20.7%)	–
Right	42(45.7%)	18 (19.6%)	24 (26.1%)	–
Age (years, mean ± SD)	62.95 ± 8.40	59.76 ± 7.93	66.58 ± 7.45	< 0.001
Height (cm, mean ± SD)	158.08 ± 7.85	159.31 ± 7.14	156.67 ± 8.45	0.113
Weight (kg, mean ± SD)	61.33 ± 10.47	60.17 ± 9.14	62.65 ± 11.78	0.26
BMI (kg/m^2^, mean ± SD)	24.51 ± 3.43	23.72 ± 3.08	25.40 ± 3.63	0.018

*n* number of participants, *non-ROA* non-radiological knee OA, *ROA* radiological knee OA, *BMI* body mass index

### Sample set division

Four features were input to model building. Thus, at least 54 participants were needed (< 92 recruited in our study). Participants were randomly divided into the training set (75%) and testing set (25%), which included 69 participants (37 non-ROA and 32 ROA) and 23 participants (12 non-ROA and 11 ROA), respectively.

### Optimal features

**Fig. 3:**
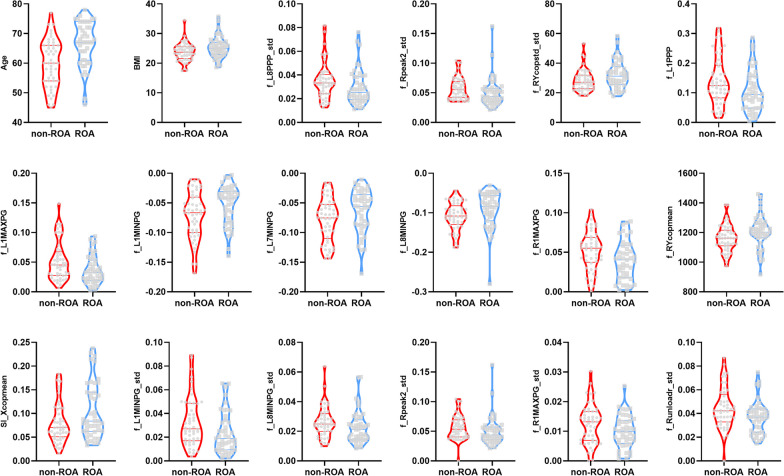
Violin diagram of the 18 features significantly differed between groups

As shown in Table 2 and Fig. 3, 18 features (2 physiological features and 16 dynamic plantar pressure features) significantly differed between groups. These plantar pressure features included the PPP, the MaxPG, the MinPG, COP displacement, SI, the second peak pressure, and the unloading rate.

**Table 2:** Significantly different features between groups

Number	Features	Mann–Whitney U	*p* value
1	f_Runloadr_std	721	< 0.001
2	Age^a^	1567.5	0.009
3	f_L8PPP_std^a^	784	0.011
4	f_Rpeak2_std^a^	759	0.013
5	f_RYcopstd_std^a^	1318	0.017
6	BMI	1341	0.02
7	f_L1PPP	781	0.021
8	f_L1MAXPG	755	0.025
9	f_L1MINPG	1329	0.029
10	f_L7MINPG	1306	0.03
11	f_L8MINPG	1371	0.031
12	f_R1MAXPG	748	0.032
13	f_RYcopmean	1380	0.033
14	SI_Xcopmean	1314	0.035
15	f_L1MINPG_std	775	0.039
16	f_L8MINPG_std	774	0.04
17	f_Lpeak2_std	791	0.042
18	f_R1MAXPG_std	779	0.049

*BMI* body mass index, *R* right, *L* left, *PPP* peak plantar pressure, *MINPG* minimum pressure gradient, *MAXPG* maximum pressure gradient, *cop* the center of pressure, *SI* symmetry index, *std* standard deviation, *peak2* the second pressure peak, *unloadr* unloading rate

^a^Optimal features included in model training

The accuracy of model was highest (Additional file 1: Fig. S1) when it considered the following four features: age, the SD of PPP in the left lateral heel (f_L8PPP_std), the SD of the right second peak pressure (f_Rpeak2_std), and the SD of variation in the anteroposterior displacement of the COP in the right foot (f_RYcopstd_std).

### Performance comparisons across models

**Fig. 4:**
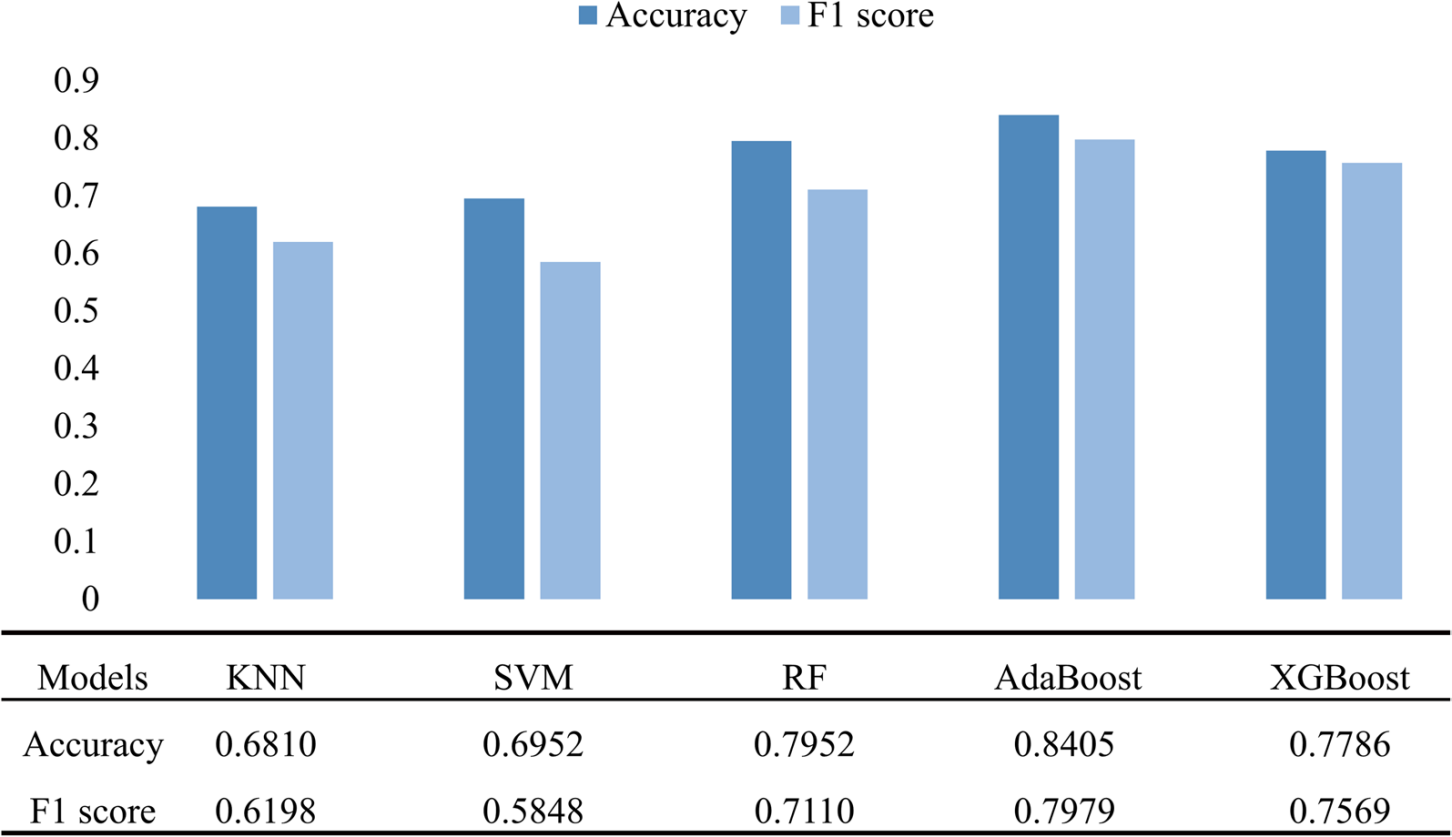
Performance of different models in the training set

**Fig. 5:**
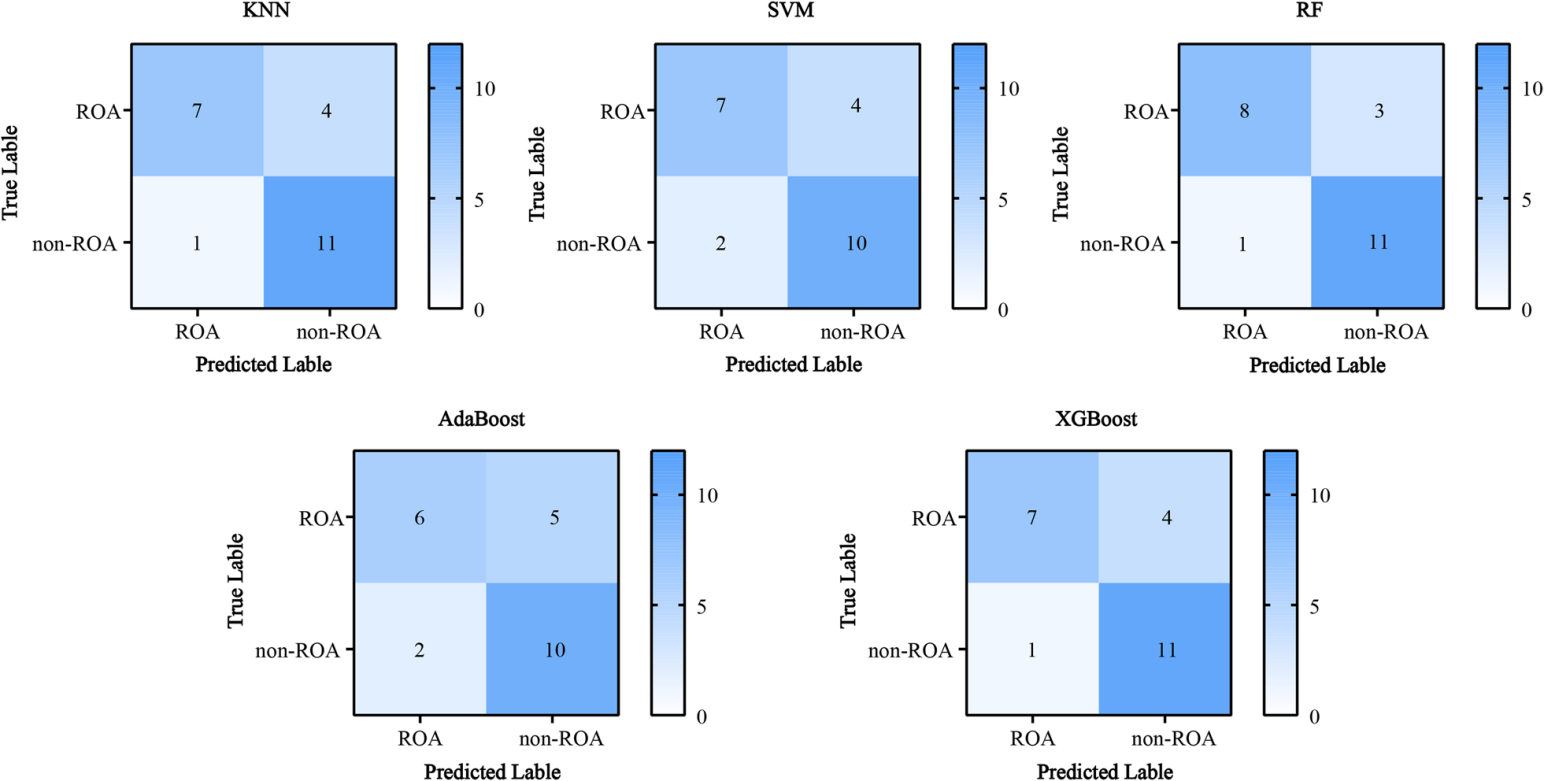
Confusion Matrices on testing set

**Fig. 6:**
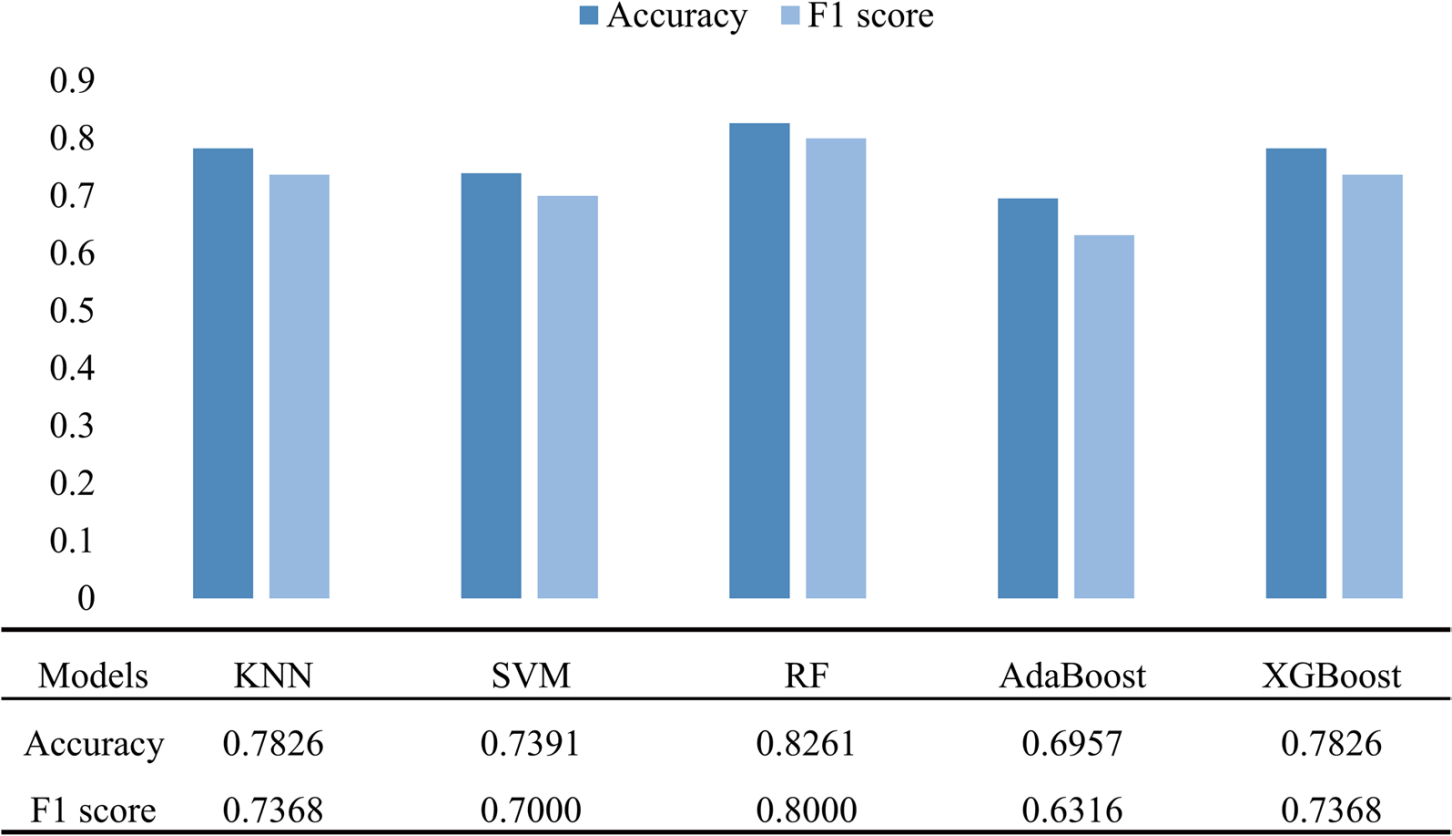
Performance of different models in the testing set

Figure 4 shows the accuracy and F1 score of each model in the training set. The accuracy and F1 score were higher than 77% and 0.7000, respectively, for the RF, AdaBoost, and XGBoost models. The testing set was applied to each model; the confusion matrix is shown in Fig. 5. The model with the best generalization ability, RF, had an accuracy of 82.61% and F1 score of 0.8000 (Fig. 6).

## Discussion

This study aimed to identify changes in dynamic plantar pressure associated with non-ROA and ROA, which was monitored dynamically using an intelligent footwear system. Machine learning models were developed to distinguish between ROA and non-ROA based on key features and showed good diagnostic performance.

Several changes in plantar pressure parameters were detected in those with ROA. As shown in Fig. 3 and Table 2**,** differences in regional plantar pressure features were primarily found on the hallux and heel, corresponding to sensor numbers 1 and 7–8, respectively. The absolute values of these features (f_L1PPP, f_L1MAXPG, f_R1MAXPG, f_R1MAXPG_std, f_L1MINPG, f_L1MINPG_std, f_L7MINPG, f_L8PPP_std, f_L8MINPG, f_L8MINPG_std) were lower in patients with ROA than in those without ROA. This finding agrees with Sito et al. [13], who reported that people with KOA had significantly lower plantar pressures on the hallux and heel as a percentage of body weight.

Four features (age, f_L8PPP_std, f_Rpeak2_std, f_RYcopstd_std) mostly associated with ROA were included in the classification models. The optimal plantar pressure features (f_L8PPP_std, f_Rpeak2_std, f_RYcopstd_std) are correlated with the standard deviation, indicating that decreased gait stability is a prominent feature of ROA. As a commonly used variable to express foot load, the PPP represents the maximum load on the foot region [36]. The PPP can be used to determine foot regions of high pressure and provides reference for the formulation of orthopedic shoes [37]. Foot orthotics are a potential simple treatment method for KOA by reducing the knee adduction moment (KAM), a mechanical biomarker of KOA progression [16]. The knee joint of individuals with ROA exhibits distinct structural changes which involve varying degrees of damage to the articular cartilage, surrounding ligaments, bursae, tendons, and muscles. Compensation of the aforementioned changes in the knee joint may occur through ankle–foot complex during walking since the lower limb functions as a kinetic chain. The f_RYcopstd_std indicates the amount of COP variation in the A/P position [38]. ROA was a key factor for unstable postural control [39]. Increased COP variability is an important predictive indicators for falls in older adults [40]. Previous study had reported that the A/P variation of COP was greater in patients with moderate KOA than that in healthy individuals [41]. An important reason of the increased A/P variation of COP in ROA group may due to the impaired knee proprioception [41]. Impaired knee proprioception will affect the motion and position sense of the lower limbs during walking, consequently reducing the balance control ability and gait stability [42]. In addition, quadriceps muscle strength shows a negative correlation with the K–L grade [43], which will in turn impact gait patterns [44]. Aging accelerating the progression of KOA due to biological alterations of knee and the cumulative effects of other risk factors is a consensus view. However, the ROA group was older than non-ROA group in this study. Therefore, findings of this study should be generalized cautiously.

Combining wearable in-shoe plantar pressure measurement devices with machine learning enables continuous and dynamic monitoring of KOA. In this way, dynamic plantar pressure can be more easily measured without being limited to test environmental conditions. Dynamic plantar pressure combined with radiography can be used to determine the KOA severity and therefore guide treatment options.

This study has several limitations that should be addressed. First, our methodology should be externally validated in a larger population to avoid potential overfitting. Moreover, the optimal plantar pressure features may be different in individuals with different affected side, but this study did not conduct a subgroup analysis on the participants based on their affected sides. Lastly, two classification grades (ROA and non-ROA) but not four-level classification (K–L grade = 1–4) were used to reflect the severity of KOA due to insufficient sample sizes.

## Conclusions

In summary, this study demonstrates that changes in dynamic plantar pressure can serve as effective mechanical biomarkers that distinguish between non-ROA and ROA employing machine learning in conjunction with an intelligent in-shoe system. Subsequent research endeavors should contemplate broader population cohorts, more classification levels, and encompass diverse functional activities to further explore the clinical potential of plantar pressure as a way to monitor the progression of KOA.

### Supplementary Information

**Additional file 1: Method S1.** Methods to calculate dynamic plantar pressure. **Table S1.** Description of modified Kellgren–Lawrence grade. **Table S2.** 210 dynamic plantar pressure features. **Fig. S1.** Optimal features selection

## Data Availability

On reasonable request, the datasets are available from the corresponding author.

## Abbreviations

BMI: Body mass index
COP: Center of pressure
DLS: Double-leg stance
KAM: Knee adduction moment
KNN: K-nearest neighbors
PG: Pressure gradient
PPP: Peak plantar pressure
PROC: Pearl River Osteoarthritis Cohort
RF: Random Forest
SD: Standard deviation
SVM: Support vector machine
SI: The symmetry index
WOMAC: The Western Ontario and McMaster Universities Osteoarthritis Index
