# Supplementary material for: Identifying changes in dynamic plantar pressure associated with radiological knee osteoarthritis based on machine learning and wearable devices
Source: J Neuroeng Rehabil. 2024 Apr 3;21:45. doi: 10.1186/s12984-024-01337-6 (PMC10988837; doi:10.1186/s12984-024-01337-6)
Supplement: Supplementary file 1 — Additional file 1: Method S1. Methods to calculate dynamic plantar pressure. Table S1. Description of modified Kellgren–Lawrence grade. Table S2. 210 dynamic plantar pressure features. Fig. S1. Optimal features selection [file 12984_2024_1337_MOESM1_ESM.docx]

**ADDITIONAL MATERIAL**

[Supplementary Method S1 Methods to calculate dynamic plantar pressure 2](#_Toc142160081)

[Supplementary Table S1 Description of modified Kellgren-Lawrence grade 4](#_Toc142160082)

[Supplementary Table S2 210 dynamic plantar pressure features 5](#_Toc142160083)

[Supplementary Figure S1 Optimal features selection 7](#_Toc142160084)

Method S1 Methods to calculate dynamic plantar pressure

The calculated methods were identical in right and left side.

1) The Peak Plantar Pressure (PPP)

- $s\in[1,8]$ represents sensor number; *L* represents the left foot; *R* represents the right foot; *n* represents sampling time point; *P_Ls_(n)* represents the pressure data of the corresponding area of each sample.

| $f\_LsPPP=\max P_{Ls}\left( n \right)$ | (1) |
| --- | --- |

- The PPP ratio between lateral heel and medial heel.

| $f\_Ls4/s2$ | (2) |
| --- | --- |

- The PPP ratio between lateral forefoot and medial forefoot.

| $f\_Ls8/s7$ | (3) |
| --- | --- |

2) The Pressure Gradient (PG)

- $\Delta t$ means the sampling time difference, the maximum PG.

| $f\_LsMaxPG=max\frac{P_{Ls}\left( n+1 \right)-P_{Ls}\left( n \right)}{\Delta t}$ | | (4) |
| --- | --- | --- |
| - The minimum PG | |  |
| $f\_LsMinPG=min\frac{P_{Ls}\left( n+1 \right)-P_{Ls}\left( n \right)}{\Delta t}$ | | (5) |
| - The loading rate | |  |
| $f\_Lloadr=max\frac{P_{L1-8}\left( n+1 \right)-P_{L1-8}\left( n \right)}{\Delta t}$ | (6) | |
| - The unloading rate |  | |
| $f\_Lunloadr=min\frac{P_{L1-8}\left( n+1 \right)-P_{L1-8}\left( n \right)}{\Delta t}$ | (7) | |

3) Time-domain Features

- The ratio between stance phase duration and swing phase duration

| $f\_Lt1/t2$ | (8) |
| --- | --- |

- The ratio between stance phase duration and gait duration

| $f\_Lt1/T\_p$ | (9) |
| --- | --- |

- The ratio between stance phase to the first PPP duration and gait cycle duration

| $f\_Lt3/T\_p$ | (10) |
| --- | --- |

- The ratio between stance phase to the second PPP duration and gait cycle duration

| $f\_Lt5/T\_p$ | (11) |
| --- | --- |

- The gait cycle duration

| $f\_LT\_p$ | (12) |
| --- | --- |

4) Impulse

- The buffer impulse of touchdown phase

| $f\_Lgbi=\sum_{n=0}^{N} \frac{P_{L1-8}\left( n \right)+P_{L1-8}\left( n+1 \right)}{2}\times\Delta t$ | (13) |
| --- | --- |

- The impulse of stance phase

| *f_Lti* | (14) |
| --- | --- |

5) The Medial-lateral Plantar Pressure Ratio (M/L)

The PPP ratio between lateral heel and medial heel.

| $f\_Ls4/s2$ | (15) | |
| --- | --- | --- |
| The PPP ratio of between lateral forefoot and medial forefoot. | |  |
| $f\_Ls8/s7$ | (16) | |

6) The Center of Pressure (COP)

| $X_{COP}=\frac{\sum_{i}^{n} F_{i}X_{i}}{\sum_{i}^{n} F_{i}}$ | (17) |
| --- | --- |
| $Y_{COP}=\frac{\sum_{i}^{n} F_{i}Y_{i}}{\sum_{i}^{n} F_{i}}$ | (18) |
| $f\_Lcopoffset =\sum_{i=0}^{N-1} \sqrt{\left( X_{i+1}-X_{i} \right)^{2}+\left( Y_{i+1}-Y_{i} \right)^{2}}$ | (19) |

7) The Symmetry Index (SI)

| $SI_{f}=\left\vert\frac{L_{f}-R_{f}}{\frac{1}{2}\left( L_{f}+R_{f} \right)} \right\vert$ | (20) |
| --- | --- |

8) Mean and standard deviation of features mentioned above during sixty gait cycles.

Table S1 Description of modified Kellgren-Lawrence grade

| Classification | Non-radiological KOA | | Radiological KOA | | |
| --- | --- | --- | --- | --- | --- |
|  | Grade 0 | Grade 1 | Grade 2 | Grade 3 | Grade 4 |
|  | normal | doubtful | mild | moderate | severe |
| Description | NO signs of OA | Suspected joint space narrowing and possible osteophyte deformation | Definite osteophytes with definite joint space narrowing | Moderate osteophytes, defined joint space narrowing, slight sclerosis, and possible articular terminal deformity | Numerous osteophytes, significant joint space narrowing, severe sclerosis, and articular terminal deformity |

Abbreviations: KOA, knee osteoarthritis

Table S2 210 dynamic plantar pressure features

| Left | Right | Symmetry index | Standard deviation  of the left | Standard deviation  of the right |
| --- | --- | --- | --- | --- |
| f_L1PPP | f_R1PPP | SI_1PPP | f_L1PPP_std | f_R1PPP_std |
| f_L1MAXPG | f_R1MAXPG | SI_1MAXPG | f_L1MAXPG_std | f_R1MAXPG_std |
| f_R1MINPG | f_R1MINPG | SI_1MINPG | f_L1MINPG_std | f_R1MINPG_std |
| f_L2PPP | f_R2PPP | SI_2PPP | f_L2PPP_std | f_R2PPP_std |
| f_L2MAXPG | f_R2MAXPG | SI_2MAXPG | f_L2MAXPG_std | f_R2MAXPG_std |
| f_R2MINPG | f_R2MINPG | SI_2MINPG | f_L2MINPG_std | f_R2MINPG_std |
| f_L3PPP | f_R3PPP | SI_3PPP | f_L3PPP_std | f_R3PPP_std |
| f_L3MAXPG | f_R3MAXPG | SI_3MAXPG | f_L3MAXPG_std | f_R3MAXPG_std |
| f_R3MINPG | f_R3MINPG | SI_3MINPG | f_L3MINPG_std | f_R3MINPG_std |
| f_L4PPP | f_R4PPP | SI_4PPP | f_L4PPP_std | f_R4PPP_std |
| f_L4MAXPG | f_R4MAXPG | SI_4MAXPG | f_L4MAXPG_std | f_R4MAXPG_std |
| f_R4MINPG | f_R4MINPG | SI_4MINPG | f_L4MINPG_std | f_R4MINPG_std |
| f_L5PPP | f_R5PPP | SI_5PPP | f_L5PPP_std | f_R5PPP_std |
| f_L5MAXPG | f_R5MAXPG | SI_5MAXPG | f_L5MAXPG_std | f_R5MAXPG_std |
| f_R5MINPG | f_R5MINPG | SI_5MINPG | f_L5MINPG_std | f_R5MINPG_std |
| f_L6PPP | f_R6PPP | SI_6PPP | f_L6PPP_std | f_R6PPP_std |
| f_L6MAXPG | f_R6MAXPG | SI_6MAXPG | f_L6MAXPG_std | f_R6MAXPG_std |
| f_R6MINPG | f_R6MINPG | SI_6MINPG | f_L6MINPG_std | f_R6MINPG_std |
| f_L7PPP | f_R7PPP | SI_7PPP | f_L7PPP_std | f_R7PPP_std |
| f_L7MAXPG | f_R7MAXPG | SI_7MAXPG | f_L7MAXPG_std | f_R7MAXPG_std |
| f_R7MINPG | f_R7MINPG | SI_7MINPG | f_L7MINPG_std | f_R7MINPG_std |
| f_L8PPP | f_R8PPP | SI_8PPP | f_L8PPP_std | f_R8PPP_std |
| f_L8MAXPG | f_R8MAXPG | SI_8MAXPG | f_L8MAXPG_std | f_R8MAXPG_std |
| f_R8MINPG | f_R8MINPG | SI_8MINPG | f_L8MINPG_std | f_R8MINPG_std |
| f_Lpeak1 | f_Rpeak1 | SI_peak1 | f_Lpeak1_std | f_Rpeak1_std |
| f_Lpeak2 | f_Rpeak2 | SI_peak2 | f_Lpeak2_std | f_Rpeak2_std |
| f_Lgbi | f_Rgbi | SI_gbi | f_Lgbi_std | f_Rgbi_std |
| f_Lti | f_Rti | SI_ti | f_Lti_std | f_Rti_std |
| f_Ls4/s2 | f_Rs4/s2 | SI_s4/s2 | f_Ls4/s2_std | f_Rs4/s2_std |
| f_Ls8/s7 | f_Rs8/s7 | SI_s8/s7 | f_Ls8/s7_std | f_Rs8/s7_std |
| f_Lloadr | f_Rloadr | SI_loadr | f_Lloadr_std | f_Rloadr_std |
| f_Lunloadr | f_Runloadr | SI_unloadr | f_Lunloadr_std | f_Runloadr_std |
| f_LXcopmean | f_RXcopmean | SI_Xcopmean | f_LXcopmean_std | f_RXcopmean_std |
| f_LXcopstd | f_RXcopstd | SI_Xcopstd | f_LXcopstd_std | f_RXcopstd_std |
| f_LYcopmean | f_RYcopmean | SI_Ycopmean | f_LYcopmean_std | f_RYcopmean_std |
| f_LYcopstd | f_RYcopstd | SI_Ycopstd | f_LYcopstd_std | f_RYcopstd_std |
| f_Lcopoffset | f_Rcopoffset | SI_copoffset | f_Lcopoffset_std | f_Rcopoffset_std |
| f_Lt1/t2 | f_Rt1/t2 | SI_t1/t2 | f_Lt1/t2_std | f_Rt1/t2_std |
| f_Lt1/T_p | f_Rt1/T_p | SI_t1/T_p | f_Lt1/T_p_std | f_Rt1/T_p_std |
| f_Lt3/T_p | f_Rt3/T_p | SI_t3/T_p | f_Lt3/T_p_std | f_Rt3/T_p_std |
| f_Lt5/T_p | f_Rt5/T_p | SI_t5/T_p | f_Lt5/T_p_std | f_Rt5/T_p_std |
| f_LT_p | f_RT_p | SI_T_p | f_LT_p_std | f_RT_p_std |

Abbreviations: R, right; L, left; std, standard deviation; PPP, peak plantar pressure; MAXPG, maximum pressure gradient; MINPG, minimum pressure gradient; peak1, the first pressure peak; peak2, the second pressure peak; gbi, buffer impulse of touchdown phase; ti, Impulse of stance phase; loadr, loading rate; unloadr, unloading rate; cop, the center of pressure; SI, symmetry index

Figure S1 Optimal features selection


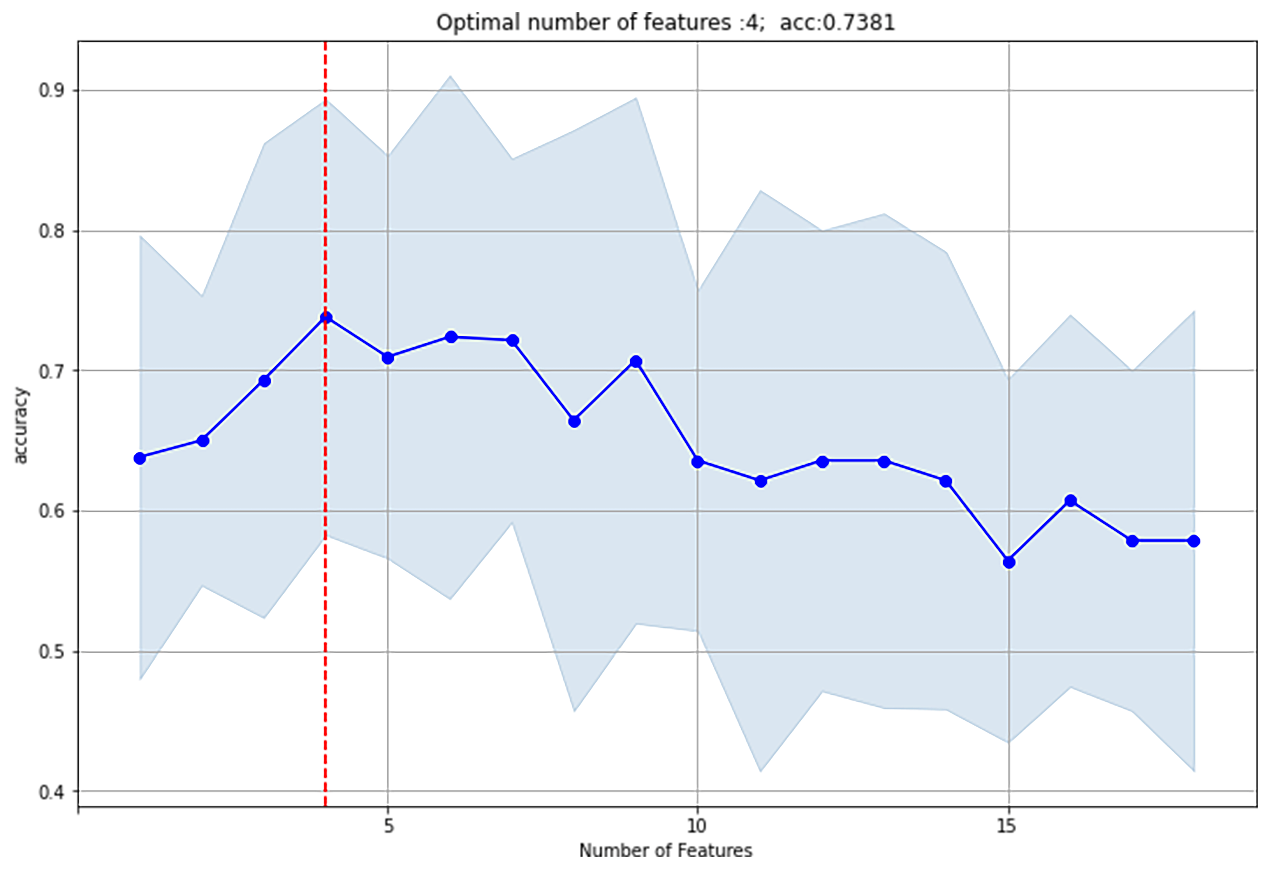


Fig. S1 Optimal features selection. The accuracy of model was highest when it considered the following four features: age, the SD of PPP of the left lateral heel (f_L8PPP_std), the SD of the right second peak pressure (f_Rpeak2_std), and the SD of the variation in the anteroposterior displacement of center of pressure (COP) in the right foot (f_RYcopstd_std).
